# Analysis of metabolic rewiring in MDR1-overexpressing drug-resistant glioblastoma

**DOI:** 10.3389/fphar.2026.1760920

**Published:** 2026-04-01

**Authors:** Manendra Singh Tomar, Ashutosh Shrivastava, Priyanka Prajapati, Chirag Kulkarni, Sreyanko Sadhukhan, Shashi Kumar Gupta, Naibedya Chattopadhyay, Amit Lahiri

**Affiliations:** 1 Center for Advance Research, Faculty of Medicine, King George’s Medical University, Lucknow, India; 2 Division of Toxicology and Experimental Medicine, CSIR-Central Drug Research Institute, Lucknow, India; 3 Academy of Scientific and Innovative Research (AcSIR), Ghaziabad, India; 4 Division of Endocrinology and Centre for Research in Anabolic Skeletal Targets in Health and Illness (ASTHI), CSIR-Central Drug Research Institute, Lucknow, India; 5 Pharmacology Division, CSIR-Central Drug Research Institute, Lucknow, India

**Keywords:** ABC transporter, glioblastoma, metabolomics, mitochondrial metabolism, temozolomide resistance

## Abstract

Glioblastoma (GBM) is a primary brain tumor, and temozolomide is the first-line alkylating agent utilized as a chemotherapeutic treatment. Despite major improvements in diagnosis and therapy, patient’s outcomes remain poor, mostly due to acquired temozolomide resistance. ABC transporter-mediated drug efflux is one of the mechanisms that play a crucial role in temozolomide resistance; however, the metabolic mechanisms that sustain MDR1 *(ABCB1)* activity in GBM remain unknown. Here, we established stable MDR1-overexpressing GBM cells and demonstrated their functional involvement in drug efflux by decreased intracellular doxorubicin accumulation and increased cell viability. Gas chromatography-mass spectrometry-based untargeted metabolomics profiling identified significantly altered metabolites in MDR1-overexpressing cells. Results of multivariate and univariate statistical analysis revealed higher levels of tricarboxylic acid (TCA) cycle intermediates that are associated with enhanced mitochondrial bioenergetics. Pathway enrichment revealed metabolite alterations in the TCA cycle, amino acid, and glutathione metabolism, indicating a coordinated metabolic rewiring potentially linked to increased ATP demand for MDR1 activity. The annexin V staining showed increased apoptotic cell populations upon metformin treatment, supporting the association between mitochondrial metabolism and intracellular drug accumulation. Overall, this study suggests that mitochondrial metabolism is a bioenergetic driver of MDR1 activity, and it could be a potential therapeutic target for overcoming MDR1-mediated drug resistance in GBM.

## 1 Introduction

Glioblastoma (GBM) is the deadliest primary brain neoplasm of the central nervous system (CNS), with a median survival rate of a little over 1 year following initial diagnosis. The incidence rate of CNS malignancies is fewer than 10 per 100,000 people, and it has been rising over the past decade (Iacob and Dinca, 2009). Standard GBM treatment involves surgery followed by radiotherapy and adjuvant chemotherapy (Hiddingh et al., 2014). The discovery of novel therapeutic regimens for GBM is largely hindered by the fact that most of the chemotherapeutic agents are unable to reach the CNS due to the blood-brain barrier (BBB). Temozolomide (TMZ) is a lipophilic imidazotetrazine molecule that may easily penetrate the BBB and thus acts as an effective therapy for gliomas (Singh et al., 2021). GBM cells can generate TMZ resistance through various mechanisms, such as drug efflux, glioma stem cells, signaling alterations, and DNA repair (Li et al., 2025). TMZ resistance in GBM is primarily caused by enhanced drug efflux via ABC transporters, with loss- and gain-of-function studies consistently confirming MDR1 (*ABCB1*) overexpression and increased activity as a significant predictor of GBM resistance to TMZ (Munoz et al., 2015). These ATP-dependent transporters actively transfer molecules, including drugs, across cell membranes against concentration gradients, regulating cellular homeostasis and contributing to drug resistance in cancer (Kathawala et al., 2015). Patients tissue examination also revealed that ABC transporters were considerably overexpressed in GBM relative to non-tumoral tissue and had a strong correlation with recurrence (Roy et al., 2022).

Numerous ABC transporters have been linked to the development of multidrug resistance in cancer, with ABCB1 (MDR1), ABCG2 (BCRP), and ABCC1 (MRP1) being the most extensively characterized (Wu et al., 2017; Sarwar et al., 2024; Teng et al., 2024). These ABC transporters require two molecules of ATP to efflux one molecule of substrate from the cells (Patzlaff et al., 2003). Regardless of the substantial energy demand required to fuel ABC transporter-mediated drug efflux, our understanding about the metabolic adaptation that supports and sustains this activity remains very limited. Thus, investigating the influence of cellular metabolism on ABC transporter activity may open up novel opportunities to develop therapies that might modulate the activity of these transporters and overcome chemoresistance.

In the present study, we overexpressed the MDR1 genes in human GBM cell lines to investigate the metabolic adaptations that influence transporter function. Numerous previous studies have revealed various metabolic pathways that support ABC transporter function in other cancers, such as mitochondrial metabolism-mediated ATP, which fuels activity in tamoxifen-resistant breast cancer cells, and glycolysis inhibition that inactivates ABC transporters and restores drug sensitivity in various cancer cells (Nakano et al., 2011; Giddings et al., 2021); however, no such study has yet been conducted in GBM. Here, we used untargeted GC-MS/MS-based metabolomic profiling to identify metabolites and metabolic pathways that were altered in GBM cells upon MDR1 overexpression. After identification, the pathway was inhibited in MDR1-overexpressing cells with metformin, and an apoptosis assay was performed. This approach allowed us to demonstrate that suppression of the metabolic pathway adversely affects cell viability in MDR1-overexpressing cells. Broadly, our data showed a potential link between metabolic reprogramming and MDR1-mediated drug resistance in GBM, suggesting metabolic pathway targeting as a possible option for impairing transporter function and overcoming chemoresistance.

## 2 Materials and methods

### 2.1 Cell culture

The human GBM cell line LN-229 was procured from the National Centre for Cell Sciences, Pune, India. Cells were cultured in Dulbecco’s Modified Eagle Medium (DMEM) supplemented with 1% non-essential amino acids, sodium pyruvate, antibiotics, and 10% fetal bovine serum (FBS). Cells were maintained at 37 °C in a humidified incubator with 5% CO_2_. Cells were subcultured at 70%–80% confluence, every 2–3 days. Cell morphology was regularly monitored by phase contrast microscopy. The culture cells were routinely checked for the presence of *mycoplasma* (Takara Bio, Japan; Cat No #RR277A).

### 2.2 Cloning of MDR1 gene

The full-length human MDR1 (*ABCB1*) gene (GenBank ID: M14758, ∼4,000 bp) was amplified by PCR using specific primers (Forward: ATT​CTC​GAG​ATG​GAT​CTT​GAA​GGG​GAC; Reverse: ATT​GGA​TCC​TCA​CTG​GCG​CTT​TGT​TCC​AG) from the pHaMDRwt (addgene, United States, Cat No #10957) plasmid. PCR amplification was carried out in a 50 µL reaction mixture containing 2 ng of template DNA, 5 µL of 10X Taq buffer, 1 µL of dNTPs, 1 µL of forward and reverse primer, 0.5 µL of Taq DNA polymerase, and nuclease-free water. The amplified PCR product was purified using the NucleoSpin Gel and PCR cleanup kit (Macherey-Nagel, Germany, 740609.50) according to the manufacturer’s protocol. The purified PCR product and plvxIRES Puro lenti expression vector were digested with BamHI (Takara, Japan, 1,605) and XhoI (Takara, Japan, Cat No #1635) restriction enzymes and resolved by agarose gel electrophoresis. The gel-purified insert and vector were ligated in a 3:1 insert-to-vector molar ratio using T4 DNA ligase (Takara, Japan, 2011A) and incubated at 16 °C for 2 h. The ligation mixture was transformed into *E. coli* DH5α competent cells. Positive clones were screened by colony PCR and confirmed by restriction digestion analysis of the recombinant plasmid.

### 2.3 Lentiviral production and transduction

A lentiviral particle carrying the MDR1 insert was prepared using HEK 293T cells, pLV x IRES Puro MDR, and lentiviral helper plasmids (psPAX2 and PMD2.G) were transfected using PEI MAX 40000 (Polyscience # 49553-93-7) in HEK293T cells. After 24 h of transfection, the medium was replaced with fresh 2.5% FBS-containing DMEM medium, and lentiviral-containing supernatant was collected at 48 h, 72 h, and 96 h post media change. The collected supernatant was precipitated with polyethylene glycol 8,000 (Sigma #P2139) and centrifuged at 2,500×g. Concentrated lentiviral particles were dissolved in 1X phosphate buffer saline. Lentiviruses containing the MDR1 gene were transduced into the LN229 cells using polybrene (10 μg/mL). Viral transduction was performed using dilutions ranging from 1:5 to 1:500 by adding 0.5 mL of viral suspension per well of a 6-well plate. Briefly, 50,000 LN-229 cells per well were introduced to virus-containing DMEM complete media supplemented with polybrene, yielding a final volume of 1.5 mL per well. Cells were treated with lentiviral particles for 48 h under conventional culture conditions. After transduction, the culture media was changed to a fresh DMEM complete medium with puromycin (5 μg/mL) to begin antibiotic selection. Selection was carried out until all untransduced cells died, which identified enriched stably transduced populations. Resistant colonies were expanded through culture to establish MDR1-overexpressing stable cell lines. The overexpression of MDR1 in the stable cells was validated by Western blot analysis. Protein extraction from cells was performed using RIPA lysis buffer with 1% protease inhibitor cocktail (HiMedia). After centrifugation at 12,000 rpm for 30 min, supernatant was collected, and protein was quantified using Bradford assay. Proteins were then prepared with 5x loading buffer, boiled at 95 °C for 10 min, and run-on 10% SDS-PAGE. After transferring to PVDF membranes and blocking with 5% BSA in TBS-Tween, membranes were incubated overnight at 4 °C with primary antibodies against MDR1 (Rabbit) (Cell signaling technology Cat No# 13978). Membrane was stripped and reprobed with GAPDH (Mouse) antibody to confirm equal loading (Abclonal Cat No #AC033). Following washes, membranes were incubated with secondary antibodies from Affinity (United States, 1:5,000), and detected using ECL (Bio-Rad).

### 2.4 Cell viability assay

The half-maximal inhibitory concentration (IC_50_) of TMZ in both control and MDR1 overexpressing LN-229 cells was determined using the MTT [3-(4,5-dimethylthiazol-2-yl)-2,5-diphenyltetrazolium bromide] assay (Amresco, United States, 0793). TMZ (TCI, Tokyo, T2744) was dissolved in dimethyl sulfoxide (DMSO) to prepare a stock solution of 150 mM. This stock was diluted in cell culture medium to achieve working concentrations for experiments. Briefly, 4 × 10^3^ cells were seeded into each well of 96-well plates and allowed to adhere before treatment with increasing concentrations of TMZ (62.5–4,000 µM). After 72 h of TMZ exposure, MTT reagent was added to each well and incubated for 4 h. The formed formazan crystals were solubilized in DMSO, and absorbance was measured at 570 nm using a microplate reader (Sharifi et al., 2019).

### 2.5 Doxorubicin (DOX) accumulation assay

Cellular retention of DOX was evaluated using flow cytometry, taking advantage of the intrinsic fluorescence of the drug. Briefly, 2 × 10^5^ cells were seeded in each well of a 6-well plate and allowed to attach overnight. The cells were then exposed to 2 μM DOX for 24 h. After treatment, cells were harvested by trypsinization, washed twice with 1X PBS to remove excess DOX, resuspended in 1 mL of PBS, and analyzed using a BD FACSLyric flow cytometer. Cells were first gated on FSC-A and SSC-A to exclude debris and dead cells, followed by FSC-A vs. FSC-H to select singlet cells. Viable singlet cells were then identified based on SSC-A vs. SSC-W parameters and intracellular DOX fluorescence was detected through the PE channel (575 nm). A total of 10,000 events were recorded for each sample. The extent of DOX accumulation was quantified as mean fluorescence intensity (MFI) (Chewchuk et al., 2018).

In a subsequent experiment, DOX accumulation in both control and MDR1-overexpressing cells was assessed alone or in the combination of metformin (500 µM) through flow cytometry as described above.

### 2.6 GC-MS/MS-based metabolite profiling

Approximately 2 million cells per sample were collected in PBS and stored at −80 °C. For metabolite extraction, 350 μL of prechilled methanol were added. The suspensions were snap-frozen in liquid nitrogen and vortexed for 2 min. The samples were then thawed at 37 °C before being centrifuged at 10,000 rpm for 10 min at 4 °C. After collecting the supernatant, the extraction process was repeated with 400 μL of cold MeOH. The supernatants were pooled after being vortexed and centrifuged under the same conditions. Next, 250 μL of ice-cold water was added and freeze-thaw cycle was repeated. After centrifugation at 10,000 rpm for 10 min, the supernatant was dried using a vacuum concentrator. Further, in each dried extract, 20 μL of methoxyamine hydrochloride (20 mg/mL in pyridine) was added and incubated at 70 °C with shaking (Thermomixer, Eppendorf; 1,100 rpm) for 90 min, converting carbonyl groups to methoximes. Subsequently, 80 μL of N-Methyl-N-(trimethylsilyl) trifluoroacetamide (MSTFA) containing 1% trimethylchlorosilane (TMCS; SRL, India, Cat. No. 83294) was added for silylation, and the reaction was carried out at 70 °C for 60 min in the Thermomixer. Derivatized samples were analyzed on a GC-MS/MS system (Thermo Trace 1300 GC coupled with TSQ 8000 MS). Quality control (QC) samples and a standard alkane mixture (Supelco, United States, Cat No #67444) were injected between the samples to monitor system performance, retention index calibration, and analytical stability. Prior to performing multivariate and univariate analyses, the MS-DIAL extracted intensities were normalized using ribitol as the internal standard, subjected to variance stabilizing transformation, and pareto scaling. Further data processing and statistical analyses were carried out using Metaboanalyst 6.0 as described earlier (Tomar et al., 2026).

### 2.7 Apoptosis assay

Annexin V-FITC/PI staining (BD Biosciences, United States) was used to measure apoptosis in accordance with the manufacturer’s instructions. A total of 1 × 10^5^ LN-229-EV/MDR1 cells were seeded. After attachment, cells were treated with TMZ (350 μM), metformin (MET) (500 μM), and their combination for 72 h. Cells were collected and resuspended in 300 µL binding buffer solution containing 5 μL Annexin V-FITC and 50 μg/mL PI dye and incubated for 30 min in the dark at 37 °C. Apoptotic cells were detected and analyzed by flow cytometry FACSLyric (BD Biosciences) (Nirwan et al., 2025). Prior to identifying separate cell populations, the gating approach was developed using forward and side scatter properties to remove debris. Then, FITC and PI fluorescence were used in quadrant analysis. For precise compensation setting and gate defining, unstained and single-stained controls were used. A total of 10,000 events were recorded for each sample. The excitation and emission wavelengths of FITC are 494 and 519 nm respectively. While the excitation and emission wavelengths of PI are 535 and 617 nm respectively.

### 2.8 Statistical analysis

All experiments were carried out in triplicate using independent samples. The results are shown as mean values ±standard deviation (SD). The IC_50_ values were calculated by nonlinear regression based on the dose-response curve generated from the absorbance data. Statistical differences between two groups were assessed using Student’s t-test. Comparisons among more than two groups were performed using one-way ANOVA followed by Tukey’s *post hoc* test. For experiments involving two independent variables, two-way ANOVA followed by Bonferroni’s *post hoc* test was applied using GraphPad Prism 5.

## 3 Results

### 3.1 Generation of MDR1-overexpressing GBM cells

**FIGURE 1:**
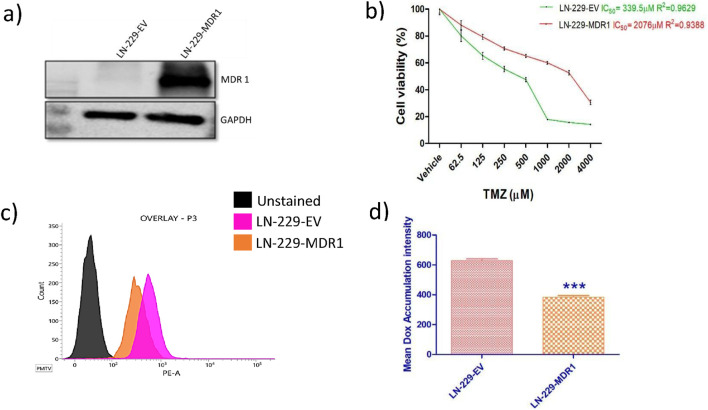
Establishing and functional characterization of MDR1-overexpressing LN-229 cells. **(a)** Western blot analysis showing relative band intensity of MDR1 expression in LN-229 cells generated through puromycin selection. GAPDH served as the internal loading control. **(b)** Effect of MDR1 expression on TMZ-mediated cell viability in LN-229 cells assessed using MTT assay. **(c)** Flow cytometry overlay showing DOX accumulation in LN-229-EV/MDR1 cells. **(d)** Quantification of Dox fluorescence in LN-229-EV and LN-229-MDR1 cells. The data are presented as the mean ± SD from three independent experiments. ***p < 0.001 compared with LN-229-EV.

LN-229 cells were transduced using lentivirus containing a human MDR1 cDNA to generate stable MDR1-overexpressing LN-229 cells. PCR amplification of the MDR1 insert resulted in a separate band of around 4.0 kb, which corresponds to the predicted size of MDR1. Colony PCR of transformants revealed positive clones, with several colonies displaying ∼4.0 kb amplicons. Restriction digestion of recombinant PLV-MDR plasmids with BamHI and XhoI released two fragments (∼8.0 kb vector backbone and ∼4.0 kb MDR1 insert), confirming correct cloning. Following lentiviral transduction, LN229 cells were subjected to puromycin selection. Untransduced control cells failed to survive antibiotic selection, while transduced cells survived and were expanded to establish a stable cell population. The stable integration of MDR1 was confirmed by Western blot analysis, demonstrating overexpression of the MDR1 protein (Figure 1a). Further, we evaluated the impact of MDR1 overexpression on drug resistance in GBM by MTT assay. Drug sensitivity was quantified by determining the half-maximal inhibitory concentration (IC_50_) of TMZ. The IC_50_ value for control LN-229-EV cells was 339.5 μM prior to MDR1 overexpression. In contrast, LN-229-MDR1 cells exhibited a markedly elevated IC_50_ of 2076 μM, indicating a substantial reduction in TMZ sensitivity (Figure 1b). The functional activity of LN-229-MDR1 cells was subjected to a DOX accumulation assay followed by flow cytometric analysis. Cells with low levels of MDR1 retained high DOX and displayed increased red fluorescence, whereas MDR1-overexpressing cells effluxed DOX effectively and showed decreased red fluorescence (Figure 1c). Quantification of DOX accumulation was performed by measuring mean fluorescence intensity (MFI) (Figure 1d). Results suggest MDR1-overexpressing cells have lower drug accumulation as well as TMZ sensitivity than the LN-229-EV phenotype.

### 3.2 Untargeted metabolomics reveals altered metabolite profile in MDR1-overexpressing LN-229 cells

**FIGURE 2:**
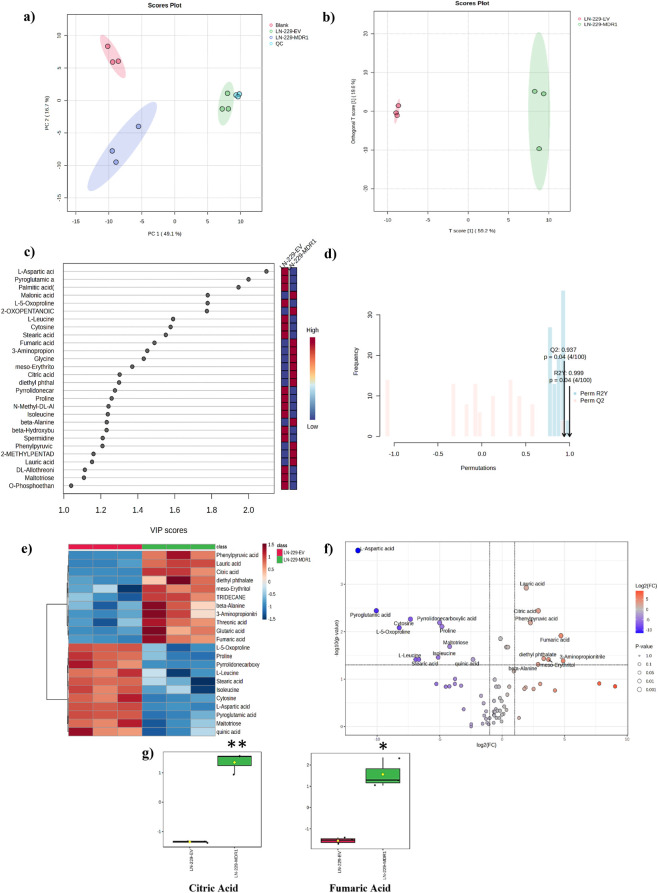
Metabolic profiling of control and MDR-1 overexpressing LN-229 cells. **(a)** The PCA plot of the metabolomics datasets comparing Blank, QC, LN-229-EV, and LN-229-MDR1. **(b)** Score plot of the OPLS-DA model showing clear separation between LN-229-EV (red circles) and LN-229-MDR1 (green circles) cells. **(c)** VIP plot displaying the 28 metabolites identified by GC–MS/MS that contributed most to group discrimination. **(d)** Model validation through 100 permutation testing, confirming the stability and robustness of the OPLS-DA model. **(e)** Heatmap showing the top 22 differentially accumulated metabolites identified by GC–MS/MS between LN-229-EV and LN-229-MDR1 cells. **(f)** Volcano plot illustrating metabolite alterations with a fold change >2.0 and an FDR-adjusted p ≤ 0.05 in MDR1-overexpressing LN-229 cells compared to LN-229-EV controls. **(g)** Box whisker plot representing higher levels of citric acid and fumaric acid in MDR1-overexpressing LN-229 cells. Statistical significance is indicated as *p < 0.05 and **p < 0.01 compared with LN-229-EV (n = 3).

We conducted multivariate exploratory analysis on 73 commonly annotated metabolites identified in both LN-229-EV and LN-229-MDR1 using a GC-MS/MS-based untargeted metabolomics approach. Multivariate analyses, including PCA and OPLS-DA, were applied to evaluate metabolite variations at both the group and individual sample levels, thereby allowing effective sample discrimination. The PCA score plot, based on the first two components, revealed a clear distinction between the LN-229-MDR1 (blue circles; n = 3), LN-229-EV (green circles; n = 3), blank (red circle; n = 3), and QC (light blue circle; n = 3). Further, separation of blanks from QC and biological samples validated the absence of systematic contamination (Figure 2a).

OPLS-DA also verified group discrimination within a 95% confidence interval, with a T-score of 59.2% and an orthogonal T-score of 19.6% (Figure 2b) (Supplementary Data S1). Based on the variable importance in projection (VIP) values, 28 metabolites were identified as important contributors to group separation (VIP score >1.0) (Supplementary Data S1). Among them, 16 metabolites (aspartic acid, pyroglutamic acid, palmitic acid, stearic acid, L-5-oxoproline, proline, leucine, cytosine, pyrrolidonecarboxylic acid, N-methyl-DL-alanine, isoleucine, beta-hydroxybutyric acid, spermidine, allothreonine, maltotriose, and o-phosphoethanolamine) were markedly reduced in LN-229-MDR1, while the remaining 12 (citric acid, fumaric acid, phenylpyruvic acid, malonic acid, 2-oxopentanoic acid, 3-aminopropionitrile, glycine, meso-erythritol, diethyl phthalate, beta alanine, lauric acid, and 2-methylpentadecanoic acid) were found at elevated levels (Figure 2c). The validity of the statistical model was confirmed using permutation and cross-validation analyses; parameters R^2^Y (0.999) and Q^2^ (0.937) suggest acceptable model performance (p-value <0.05) (Figure 2d).

Univariate statistical analysis through student’s t-test suggests that 22 out of the 73 metabolites were significantly altered in MDR1-overexpressing LN-229 cells (p ≤ 0.05 and FDR ≤0.05). Among them, 11 metabolites were reduced (positive t-stat values), while 11 displayed elevated levels (negative t-stat values) (Figure 2e) (Supplementary Data S1). Notably, TCA cycle intermediates such as citric acid and fumaric acid were up accumulated. In addition, beta alanine, an amino acid that is involved in cellular energy support, is also reported up accumulation in MDR1-overexpressing cells. The results of the volcano plot analysis showed that 8 metabolites were significantly up-accumulated, whereas 11 were down-accumulated in MDR1-overexpressing LN-229 cells. These findings demonstrated that the TCA cycle metabolites citric acid and fumaric acid had over twofold accumulation in LN-229-MDR1 cells (Figure 2f) (Supplementary Data S1). These important TCA cycle intermediates were represented as box-and-whisker plots (Figure 2g).

### 3.3 Metabolite pathway enrichment and biomarker analysis

**FIGURE 3:**
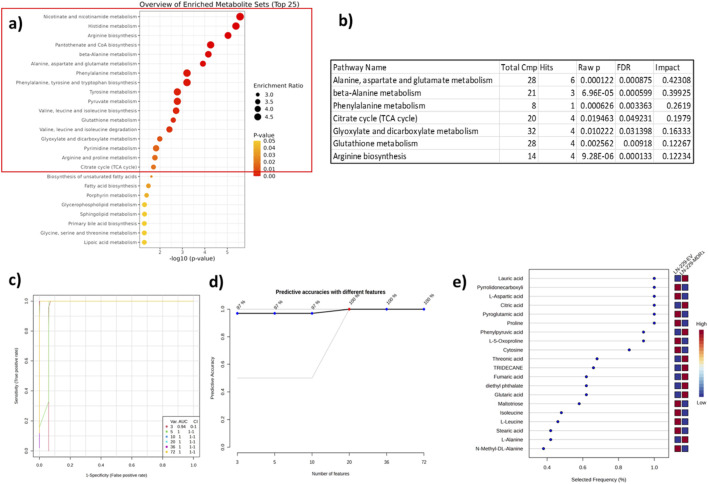
Metabolic pathway enrichment and SVM-based classification in MDR-1 overexpressing cells. **(a)** The plot highlights the top 25 most enriched and physiologically relevant metabolic pathways associated with MDR1 overexpression in GBM (p-value ≤0.05). **(b)** Key metabolic pathways altered significantly in association with MDR1-mediated drug resistance in GBM (p-value ≤0.05 and Impact >0.1). **(c)** Model performance test for SVM classifiers with a continuous increase in the number of metabolites. ROC curve of each SVM classifier based on the average cross-validation performance. The AUC and 95% CI are displayed in the panel. **(d)** Predictive accuracy with different features for the different SVM classifiers. The model shows the highest accuracy with 20 metabolites highlighted in red. **(e)** The 20 most important variables for the classification of LN-229-EV and LN-229-MDR1 cells.

The Kyoto Encyclopedia of Genes and Genomes (KEGG) database was used to conduct enrichment analysis to identify metabolic pathways that had been significantly altered in MDR1-overexpressing LN-229 cells. The top 25 enriched metabolic pathways were ranked according to statistical significance and enrichment ratio; the red line highlights the pathways that have an FDR-corrected p-value ≤0.05 (Figure 3a) (Supplementary Data S1). Further, we performed pathway analysis that identified seven metabolic pathways impacted significantly, encompassing key nodes of central carbon metabolism, amino acid metabolism, and redox homeostasis (FDR-corrected p-value ≤0.05 and impact >0.1) (Figure 3b). In particular, the TCA cycle was identified as one of the most significantly altered, with corresponding metabolites displaying elevated accumulation in LN-229-MDR1 cells. In addition to energy metabolism, other amino acid-related pathways were considerably enriched, including alanine, aspartate, and glutamate metabolism; β-alanine metabolism; phenylalanine metabolism; and arginine biosynthesis. These pathways are directly associated with anaplerotic TCA cycle replenishment, nitrogen balancing, and biosynthetic activities, demonstrating a coordinated metabolic response that promotes long-term cell growth and survival (Chandel, 2021). Additionally, glutathione metabolism was considerably altered in MDR1-overexpressing cells, indicating an improved antioxidant defense system. While maintaining the high ATP requirement and survival advantage associated with MDR1-mediated drug resistance.

Moreover, biomarker analyses were carried out to identify the minimal number of metabolites capable of illustrating and interpreting the difference between MDR1-overexpressing and control LN-229 cells. In this study, the support vector machine (SVM) model was constructed using a progressively increasing number of metabolite features. Using three metabolites, the SVM model had good classification performance with an AUROC of 0.94 (95% CI = 0-1). Increasing the number from 5 metabolites to 72, model performance improved, and AUROC reached 1.0 (95% CI = 1-1) (Figure 3c). Similar results were observed on predictive accuracies, where a lower feature number yielded slightly reduced accuracy, while the addition of 17 metabolites increased the accuracy by 100%, that is remained constant upon reaching a number of 72 features (Figure 3d). Based on model performance and feature stability, a panel of 20 metabolites was selected as the final feature set for the SVM model. Among these 20 important metabolite features, 18 were identified as statistically significant in the t-test analysis in the LN-229-MDR1 overexpressing (p-value ≤0.05). Interestingly, some of the statistically nonsignificant (p-value >0.05) metabolites were also selected, namely, L-alanine and N-methyl DL-alanine, indicating that these metabolites contribute to multivariate discrimination despite modest individual effects (Figure 3e). Given the limited number of independent eplicates (n = 3 per group), these results should be interpreted as exploratory and indicative of candidate metabolic biomarkers rather than definitive diagnostic markers.

### 3.4 Determine the significance of mitochondrial metabolism in cellular sensitization

**FIGURE 4:**
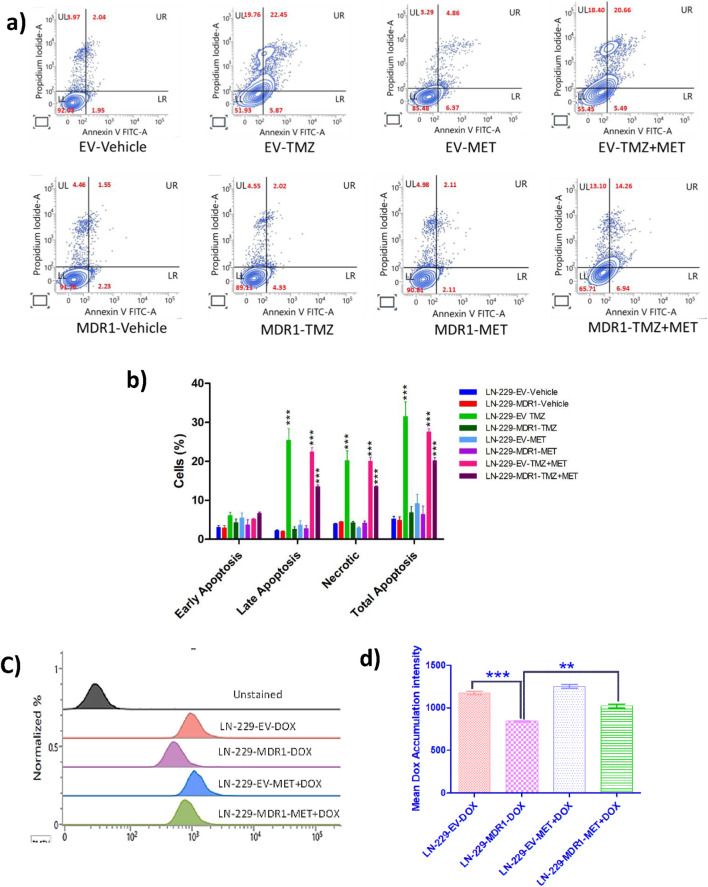
Metformin enhances TMZ-induced cell death in MDR1-overexpressing GBM. **(a)** Representative flow cytometric dot plots of Annexin V-FITC/propidium iodide (PI) staining in LN-229-EV and MDR1-overexpressing cells after treatment with VEH, TMZ (350 µM), MET (500 µM), and MET + TMZ. **(b)** Quantitative plot of % apoptotic and necrotic cells. **(c)** Dox accumulation assay to assess the impact of metformin on MDR1-mediated drug efflux. Cells were treated with 500 µM of MET for 24 h. **(d)** Quantification of mean Dox fluorescence intensity in LN-229-EV and LN-229-MDR1 cells after MET treatment. Data are presented as mean ± SD from at least three independent experiments. Statistical significance is indicated as **p < 0.01, and ***p < 0.001 (n = 3).

To evaluate the involvement of mitochondrial metabolism in cellular sensitization, apoptosis was assessed by Annexin V-FITC/propidium iodide (PI) flow cytometry. TMZ treatment induced cytotoxicity in control cells and failed to elicit a significant apoptotic response in MDR1-overexpressing cells. Alone, metformin (MET) did not have a significant impact on cell death, but in combination with TMZ (MET + TMZ), it had a pronounced increase in late apoptotic and necrotic populations, particularly in MDR1 cells, reflecting a synergistic enhancement of apoptosis. Collectively, these findings demonstrate that MET treatment is consistent with inhibition of mitochondrial metabolism and sensitization to TMZ, with the combination treatment (MET + TMZ) exhibiting the strongest pro-apoptotic effect (Figures 4a,b). Furthermore, the influence of metformin-mediated mitochondrial metabolism suppression on MDR1-mediated drug efflux was investigated using a DOX accumulation assay. DOX accumulation results in an increase in mean fluorescence intensity in metformin-treated MDR1 cells compared to non-treated (Figures 4c,d). These findings clearly indicate that inhibiting mitochondrial metabolism affects the activity of MDR1-overexpressing GBM cells, resulting in enhanced drug accumulation and cytotoxicity.

## 4 Discussion

There are various mechanisms of TMZ resistance in GBM, among which drug efflux by ABC transporters like MDR1 is one of the most important mechanisms permitting tumor survival under chemotherapeutic therapy (Radtke et al., 2022). The function of MDR1-mediated drug efflux in drug resistance has been extensively identified across numerous malignancies (Skinner et al., 2023); the metabolic factors that drive ABC transporter activity in GBM remain largely unknown. Recent studies have also underlined the importance of metabolic reprogramming in TMZ-resistant GBM cells, since it improves both energy generation and cell survival. Key metabolic alterations in TMZ-resistant GBM cells include increased Warburg effect, TCA cycle, fatty acid oxidation, glutamine metabolism, and antioxidant defense-related metabolites. These pathways contribute to drug efflux, counteract drug-induced oxidative stress, and promote drug resistance (Tsai et al., 2022; Tomar et al., 2026; Yan et al., 2026). Similarly, rewiring of purine metabolism is a known key factor in TMZ resistance, whereas guanosine and inosine can serve as adjuvants to increase cytotoxicity in resistant cells (D’Aprile et al., 2025). The results of the DOX accumulation experiment show that MDR1-overexpressing cells have lower DOX accumulation and display drug resistance in GBM. Then we used untargeted metabolomics to discover the metabolic alterations required to meet the bioenergetic requirement for MDR1 activity. Metabolic rewiring, particularly TCA cycle augmentation as well as alteration in amino acid and glutathione metabolism, was reported in MDR1-overexpressing cells. These results are consistent with fundamental biochemical principles governing ABC transporters that suggest substantial ATP hydrolysis is required to drive the efflux of drugs against concentration gradients (Patzlaff et al., 2003). MDR1-overexpressing LN-229 cells had increased TCA cycle intermediates, particularly citrate and fumarate, indicating increased mitochondrial activity to fulfill the high ATP requirement for MDR1-mediated drug efflux. Earlier studies in breast, myeloma, ovarian, hepatocellular, and lymphoma models showed that increased glycolysis or oxidative phosphorylation (OXPHOS) supports ABC transporter-mediated drug resistance, but no such investigation has been conducted in GBM (Nakano et al., 2011; Xintaropoulou et al., 2018; Giddings et al., 2021).

In addition to energy metabolism, pathway enrichment analysis showed that amino acid metabolic pathways such as alanine, aspartate, glutamate, phenylalanine, and beta-alanine metabolism were significantly influenced. Amino acids are crucial substrates for both bioenergetic and biosynthetic activity in highly proliferative cells, since their carbon skeleton feeds into metabolic pathways to create ATP, glucose, and fatty acids (Chen and Russo, 2012; Phan et al., 2014; Chandel, 2021). Glutamate, a major anaplerotic substrate, replenishes α-ketoglutarate and maintains TCA cycle flow (Andersen, 2025). Similarly, alanine contributes to TCA cycle metabolites (e.g., citrate) and contributes to transaminase products (e.g., glutamate, aspartate, proline, serine) (Parker et al., 2020). Phenylalanine metabolism also favors rapid growth and survival, primarily by catabolism into nutrients that feed the TCA cycle (Cheng et al., 2024). Upregulation of these pathways in MDR1-overexpressing cells suggests a possible coordinated metabolic program aimed at maximizing mitochondrial function.

MDR1-overexpressing cells had higher levels of beta-alanine, which is a metabolic sign of increased energy buffering ability and mitochondrial respiration (Schnuck et al., 2016). In GBM cells, the glutathione metabolism reflects enhanced antioxidant defense, which is a common feature in drug-resistant cancer cells (Hammoudi et al., 2011). Increased glutathione turnover allows cells to reduce oxidative stress caused during chemotherapy and promotes cell survival during TMZ exposure (Rocha et al., 2016; Niu et al., 2021). Our data also show substantial decreases in numerous amino acids and fatty acids in MDR1-overexpressing cells. Decreased abundance of amino acids such as aspartic acid, pyroglutamic acid, proline, leucine, and isoleucine may be associated with TCA cycle anaplerosis or stress-induced biosynthetic processes. Reduced levels of fatty acids, including palmitic and stearic acid, were identified in multivariate statistical analysis, suggesting a shift away from lipid synthesis toward oxidation pathways that generate reducing equivalents and fuel mitochondrial ATP production. This metabolic redirection highlights the extensive remodeling occurring in resistant GBM cells to maintain MDR1 activity.

Increased TCA cycle flux provides abundant ATP for MDR1 phosphorylation and substrate binding, essential steps for drug efflux (Hagen et al., 2022). Such metabolic flexibility is consistent with previous studies showing that therapy-resistant tumors exhibit greater adaptability in energy metabolism, enabling them to withstand chemotherapeutic-induced stress (Nakahara et al., 2023). These results suggest targeting mitochondrial bioenergetics could represent a promising strategy to overcome MDR1-mediated resistance. In fact, combining the mitochondrial inhibitor metformin with TMZ induces cell death and restores cellular sensitization in MDR1-overexpressing cells.

Despite the strengths of this study, certain limitations must be acknowledged. Untargeted metabolomic analyses provide only semi-quantitative information and are inherently limited by factors such as incomplete metabolite coverage and reliance on spectral libraries for compound annotation. Consequently, the results obtained from untargeted workflows should be interpreted as exploratory and hypothesis-generating rather than conclusive. To confirm the biological relevance of the metabolic changes observed in our study, targeted metabolomics will be necessary, as it enables absolute quantification with greater specificity and sensitivity. Complementing these analyses with an orthogonal platform such as liquid chromatography-mass spectrometry (LC-MS) could strengthen metabolite identification and extend pathway coverage, including those pathways potentially associated with TMZ resistance. The work was conducted in a single GBM cell line model, and further validation in additional GBM models, including patient-derived cells and *in vivo* systems, will be required.

In conclusion, our data show that mitochondrial metabolism is critical in MDR1-mediated drug resistance in GBM. These findings point to metabolic pathways, specifically the TCA cycle and amino acid metabolism, as prospective therapeutic targets. Future research should look into metabolic inhibitors and combination techniques to limit MDR1 function and boost chemotherapeutic efficacy in GBM.

## Data Availability

The raw data supporting the conclusions of this article will be made available by the authors, without undue reservation.
